# Impact of Restricting Visitors to Nursing Homes During the Pandemic: Causal Inferences and Qualitative Analysis

**DOI:** 10.1093/geroni/igaf122.1942

**Published:** 2025-12-31

**Authors:** Huiwen Xu, Edward Miller

## Abstract

Early in the COVID-19 pandemic, federal and state governments implemented visitation restrictions to nursing homes to reduce the risk of infections. However, those restrictions also removed a significant amount of personal care and social support provided by family and friends of nursing home residents. Later in 2020, these restrictions were lifted. In this symposium, we leveraged unique visitation data and family satisfaction surveys, and novel causal inference methods to understand the impact of visitation restrictions on resident infections and quality of life. The first study analyzed facility-level visitation data to examine whether allowing visitors was associated with increased resident COVID-19 infection rates. After weighting facilities by the inverse of the probability of allowing visitors, we found no relationship between nursing homes’ allowing visitors and COVID-19 infections among their residents. The second study utilized a difference-in-differences event study framework to compare changes in nursing home COVID-19 infection rates in the 4 weeks before vs. 8 weeks after the lifting of the federal visitation restriction on September 17, 2020. Study results suggest that lifting the federal visitation restriction had a negligible impact on nursing home infection rates. The third study qualitatively analyzed open-ended comments in a statewide family satisfaction survey in Ohio to identify the impacts of visitation bans. The majority of survey commenters reported negative social and emotional impacts on both residents and family members. In summary, allowing visitors back did not increase the risk of COVID-19 infections, but may improve quality outcomes for residents and family members.

